# Eighty phenomena about the self: representation, evaluation, regulation, and change

**DOI:** 10.3389/fpsyg.2015.00334

**Published:** 2015-03-27

**Authors:** Paul Thagard, Joanne V. Wood

**Affiliations:** ^1^Department of Philosophy, University of Waterloo, Waterloo, ON, Canada; ^2^Department of Psychology, University of Waterloo, Waterloo, ON, Canada

**Keywords:** self, mechanisms, self-change, self-representation, self-regulation

## Abstract

We propose a new approach for examining self-related aspects and phenomena. The approach includes (1) a taxonomy and (2) an emphasis on multiple levels of mechanisms. The taxonomy categorizes approximately eighty self-related phenomena according to three primary functions involving the self: representing, effecting, and changing. The representing self encompasses the ways in which people depict themselves, either to themselves or to others (e.g., self-concepts, self-presentation). The effecting self concerns ways in which people facilitate or limit their own traits and behaviors (e.g., self-enhancement, self-regulation). The changing self is less time-limited than the effecting self; it concerns phenomena that involve lasting alterations in how people represent and control themselves (e.g., self-expansion, self-development). Each self-related phenomenon within these three categories may be examined at four levels of interacting mechanisms (social, individual, neural, and molecular). We illustrate our approach by focusing on seven self-related phenomena.

## Introduction

Social and clinical psychologists frequently use the concept of the self in their discussions of a wide range of phenomena (e.g., Baumeister, 1999; Sedikides and Brewer, 2001; Leary and Tangney, 2003; Alicke et al., 2005; Sedikides and Spencer, 2007). However, there is no general, unified psychological theory of the self that can account for these phenomena. Thagard (2014) has proposed a view of the self as a multilevel system consisting of social, individual, neural, and molecular mechanisms. Like James (1890) and Mead (1967), this view accommodates social, cognitive, and physiological aspects of the self, but provides far more detail about the nature of the relevant mechanisms. Our aim in the current paper is to show the applicability of the multilevel system account of the self to a large range of phenomena.

We will present a new taxonomy that categorizes approximately eighty self-related phenomena according to three primary aspects of the self: representing, effecting, and changing. The representing self encompasses the ways in which people depict themselves, either to themselves or to others (e.g., self-concepts, self-presentation). The effecting self concerns ways in which people facilitate or limit their own traits and behaviors (e.g., self-enhancement, self-regulation). The changing self is less time-limited than the effecting self; it concerns phenomena that involve lasting alterations in how people represent and control themselves (e.g., self-expansion, self-development). After presenting this taxonomy, we will describe how four levels of mechanisms—social, individual, neural, and molecular—are relevant to understanding these phenomena about the self. It would be premature to offer a full theory of the self, because not enough is known about the nature of these mechanisms and how they produce the relevant phenomena. But we hope our taxonomy and outline of relevant mechanisms provides a new and useful framework for theorizing about the self.

## A Taxonomy of Self-Phenomena

There are more than eighty frequently discussed topics that we call the self-phenomena. More accurately, each of these topics should be understood as a group of phenomena. For example, there are many empirical findings about self-esteem that should count as distinctive phenomena to be explained, so there are potentially hundreds of findings for which a scientific theory of the self should be able to account.

**FIGURE 1:**
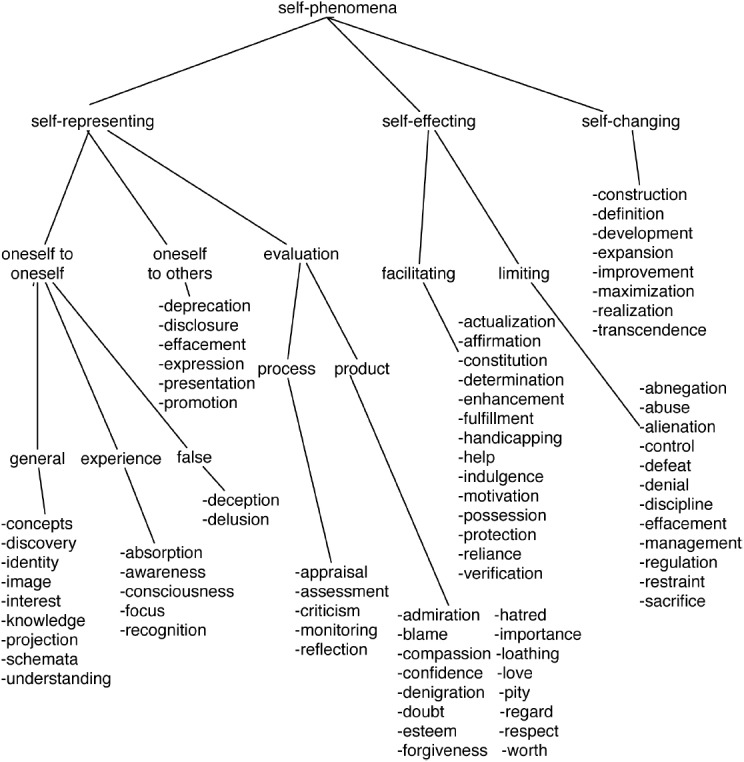
**Grouping of many self-phenomena into six main classes: self-representing (with three sub-categories), self-effecting (with two sub-categories), and self-changing.** Source: Thagard (2014).

Fortunately, the task of accounting for all of the self-phenomena, through causal explanations of the large number of empirical findings about them, can be managed by grouping the phenomena according to three primary aspects of the self: representing, effecting, and changing. All of the self-phenomena fall primarily under one of these functional groups, although a few are related to more than one group. Figure 1 summarizes the proposed organization of self-phenomena that we now discuss in more detail.

### The Representing Self

A representation is a structure or activity that stands for something, and many of the self-phenomena listed in Figure 1 concern ways in which people represent themselves. The representing self can roughly be divided into three subgroups concerned with (1) depicting oneself to oneself, (2) depicting oneself to others, and (3) evaluating oneself according to one’s own standards.

The most general terms for depicting oneself to oneself are self-knowledge and self-understanding, which seem roughly equivalent. Self-concepts and self-schemata are both mental ingredients of self-knowledge, serving as cognitive structures to represent different aspects of the self. (Later we provide a more detailed account of self-concepts.) Self-interest consists in the collection of one’s personal goals, conscious or unconscious. Self-identity and self-image are also ways in which one represents oneself to oneself, although they may also contribute to how one represents oneself to others. Self-discovery and self-projection are processes that involve self-representation.

Several aspects of depicting oneself to oneself assume conscious experience, as in self-awareness and other phenomena listed in Figure 1. Such experience is not purely cognitive, as it can also involve prominent affective components such as moods and emotions. Another set of phenomena that involve depicting oneself to oneself includes self-deception and self-delusion, in which the representation of self is false. The second division within the group of self-representing phenomena involves depicting and communicating oneself to others.

The third sub-group of self-phenomena in the representing category concerns the evaluation of the self, either as on ongoing process or as the product that results from the evaluation. Phenomena concerned with the process of evaluation include self-appraisal. There are many products that result from this process, including both general assessments such as self-confidence and particular emotional reactions such as self-pity.

### The Effecting Self

The self does more than just represent itself; it also does things to itself, including facilitating its own functioning in desirable ways and limiting its functioning to prevent undesirable consequences. Self-phenomena that have a facilitating effect include self-actualization. Self-evaluation can also produce the self-knowledge that unconstrained actions may have undesirable consequences, as in excessive eating, drinking, drug use, and dangerous liaisons. Accordingly, there is a set of important phenomena concerning limits that people put on their own behavior, including self-control. All of these self-effecting phenomena involve people encouraging or discouraging their own behaviors, but they do not bring about fundamental, longer lasting changes in the self, which is the third and probably rarest aspect of the self.

### The Changing Self

Over a lifetime, people change as the result of aging and experiences such as major life events. Some self-phenomena such as self-development concern processes of change. The changes can involve alterations in self-representing, when people come to apply different concepts to themselves, and also self-effecting, if people manage to change the degree to which they are capable of either facilitating desired behaviors or limiting undesired ones. Whereas short-term psychotherapy is aimed at dealing with small-scale problems in self-representing and self-efficacy, long-term psychotherapy may aim at larger alterations in the underlying nature of the self.

The proposed grouping of self-phenomena summarized in Figure 1 is not meant to be exhaustive, as there are aspects of self that are described by words without the “self” prefix, such as agency, autonomy, personhood, and resilience, as well as more esoteric terms that do use the prefix. But the diagram serves to provide an idea of the large range of phenomena concerning the self. Our goal is to show the applicability of the multilevel account of the self to this range of phenomena, by selecting phenomena from each of the six main classes in Figure 1. It would be tedious to apply the multilevel theory to more than eighty phenomena, so we take a representative sampling that includes: self-concepts, self-presentation, self-esteem, self-enhancement, self-regulation, self-expansion, and self-development. Each of these has aspects that need to be understood by considering the self as a system that operates at social, individual, neural, and molecular levels.

**FIGURE 2:**
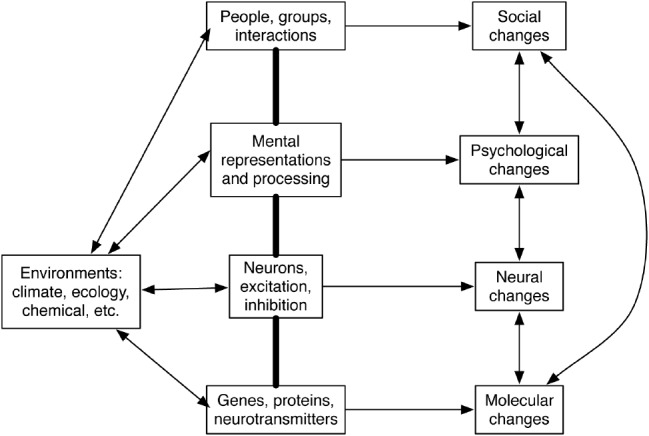
**Diagram of the self as a multilevel system.** Lines with arrows indicate causality. Thick lines indicate composition. Source: Thagard (2014).

Figure 2 displays the relevant levels and their interconnections. We understand a mechanism to be a system of parts whose interactions produce regular changes (Bechtel, 2008; Thagard, 2012). The social level consists of people who communicate with each other. The individual level consists of mental representations and computational procedures that operate on them. The neural level consists of neurons that excite and inhibit each other. Finally, the molecular level consists of genes, proteins, neurotransmitters, and hormones that affect neural operation. For defense of this account of levels of mechanisms, and the occurrence of causal links between social and molecular levels, see Thagard (2014).

We do not mean to suggest that there are three separate selves capable of representing, effecting, and changing, any more than we implied that there are separate social, individual, neural, and molecular selves. We especially want to avoid the ridiculous suggestion that a person might consist of twelve different selves combining three different aspects at four different levels. Our goal is to display the unity of the self, not just its amazing diversity. Unification arises first from seeing the interconnections of the four levels described earlier, and second from recognizing how the interconnected mechanisms produce all three of the self’s functions.

## The Representing Self

The scientific value of understanding the self as a multilevel system depends on its fruitfulness in generating explanations of important empirical findings concerning the various self-phenomena. We will attempt to show the relevance of multiple mechanisms for understanding three phenomena that are involved in representational aspects of the self: self-concepts, self-presentation, and self-esteem. Respectively, these involve representing oneself to oneself, representing oneself to others, and evaluating oneself.

### Self-Concepts (Representing Oneself to Oneself)

Self researchers distinguish between self-concept, which involves *content*—one’s thoughts, beliefs, and knowledge about the self—and self-esteem, which involves *evaluation*—evaluation of oneself as good, bad, worthy, unworthy, and so forth. Here we focus on self-concepts, considering them at individual, social, neural, and molecular levels. Psychologists studying the self no longer think of people as possessing a single, unified self-concept, but as possessing self-views in many domains (Baumeister, 1999). People have various concepts that they apply to characterize themselves with respect to features such as gender, race, ethnicity, nationality, religion, occupation, hobbies, personality, and physical characteristics. For example, a man might think of himself as an intellectual, Canadian, and aging father. Moreover, not all content of those various self-views can be held in mind at once. The part of self-concept that is present in awareness at a given time has been called the “working self-concept” (Markus and Kunda, 1986). What is the nature of the concepts that people apply to themselves, and what are the mechanisms underlying these applications?

The individual level of mental representations is clearly highly relevant to understanding concepts including ones about the self. What kind of mental representations are concepts? Unfortunately, there is no single currently available psychological theory of concepts that can be applied to self-concepts. Debate is ongoing about whether concepts should be understood as prototypes, collections of exemplars, or theoretical explanations (Murphy, 2002; Machery, 2009), and all of these aspects are relevant to self-concepts (Kunda, 1999, Ch. 2). For example, the concept of extravert carries with it prototypical conditions such as enjoying social interactions, exemplars such as Bill Clinton, and explanations such as people going to parties *because* they are extraverted. Below we will suggest how all of these aspects of concepts can be integrated at the neural level.

Psychological mechanisms such as priming carried out by spreading activation between concepts explain how different concepts get applied in different situations. For example, people at parties may be especially prone to think of themselves as extraverted. Such explanations require also taking into account social mechanisms such as communication and other forms of interaction. Then the causes of applying the concept *extraverted* to oneself include social mechanisms as well as the individual mechanism of spreading activation among concepts.

The vast literature on self-concepts points to the interplay of the individual and social levels in a myriad of ways. First is research on social comparison, which shows that one’s working self-concept depends on the other people present (Wood, 1989). Ads with skinny models can make one feel fat, and unkempt people can make one feel well-groomed. When asked to describe themselves, people tend to list characteristics that make them distinctive in their immediate social setting. A woman in a group of men is especially likely to list her gender, and a white man in a group of African–American men is especially likely to list his race (e.g., McGuire et al., 1978).

More permanent aspects of one’s social surround can have more consequential effects on self-concept. For example, college graduates’ career aspirations depend on their standing relative to their peers at their own college, regardless of the college’s standing relative to other institutions (Davis, 1966). A student who earns high grades at institutions where grading is easier tends to have higher career aspirations than an equally qualified student at a more competitive college. This phenomenon has been called “the campus as a frog pond”; for the frog in a shallow pond aims his [or her] sights higher than an equally talented frog in a deep pond (Pettigrew, 1967, p. 257). According to social identity theory, one psychological basis of group discrimination is that people identify with some groups and contrast themselves with other groups that are viewed less favorably (Tajfel, 1974).

Self-concepts are also influenced by the culture in which one lives. Markus and Kitayama (1991) proposed that whereas Westerners have more “independent self-construals,” in which the self is autonomous and guided by internal thoughts and feelings, Asians have more “interdependent self-construals,” in which the self is connected with others and guided, at least in part, by others’ thoughts and feelings.

Another way that the individual and social levels intersect with respect to self-concept involves the “looking-glass self” or “reflected appraisals”—the idea that people come to see themselves as others see them. This idea has been prominent in social science for some time (e.g., Mead, 1967), but research in social psychology in the last few decades leads to a different conclusion: People do not see very clearly how others, especially strangers, see them, and instead believe that others see them as they see themselves (see Tice and Wallace, 2003, for a review). Instead of others’ views influencing one’s self-view, then, one’s self-view determines how one thinks others view oneself. It is possible, however, that within close relationships, the reflected self plays a greater role in shaping the self-concept (Tice and Wallace, 2003).

Feedback from others can also affect self-concepts, and not just in the way one might expect. For example, although people may think of themselves as more attractive when they have been told they are attractive, people sometimes resist others’ feedback in various ways (Swann and Schroeder, 1995). For example, when people with high self-esteem (HSEs) learn they have failed in one domain, they recruit positive self-conceptions in other domains (e.g., Dodgson and Wood, 1998). People are more likely to incorporate others’ feedback into their self-views if that feedback is close to their pre-existing self-view than if it is too discrepant (Shrauger and Rosenberg, 1970).

Self-concepts also change with one’s relationships. Two longitudinal studies showed that people’s self-descriptions increased in diversity after they fell in love; people appear to adopt some of their beloved’s characteristics as their own (Aron et al., 1995). Several studies also indicate that cognitive representations of one’s romantic partner become part of one’s own self-representation (as reviewed by Aron, 2003). Andersen and Chen (2002) describe a “relational self” in which knowledge about the self is linked with knowledge about significant others.

Interactions with other people also affect the self-concept through a process called “behavioral confirmation,” whereby people act to confirm other people’s expectations (Darley and Fazio, 1980). For example, when male participants were led to believe that a woman they were speaking to over an intercom was physically attractive, that woman ended up behaving in a more appealing way than when the man thought she was unattractive (Snyder et al., 1977). Presumably, a man’s expectation that a woman is attractive leads him to act especially warmly toward her, which in turn brings to the fore a working self-concept for her that is especially friendly and warm. Evidence suggests that when people believe that others will accept them, they behave warmly, which in turn leads those others to accept them; when they expect rejection, they behave coldly, which leads to less acceptance (Stinson et al., 2009). More consequential results of behavioral confirmation are evident in a classic study of the “Pygmalion” effect, in which teachers were led to have high expectations for certain students (randomly determined), who then improved in academic performance (Rosenthal and Jacobson, 1968).

So far we have considered social effects on the self-concept. In turn, one’s self-concept influences one’s judgments of others in many ways. In his review of this large literature, Dunning (2003) grouped such effects into three main categories. First, in the absence of information about others, people assume that others are similar to themselves. Second, in their impressions of another person, people emphasize the domains in which they themselves are strong or proficient. Third, when judging others on some dimension, such as physical fitness, people tend to use themselves as a benchmark. Given a man who takes a daily 20-min walk, athletes will judge him to be unfit, whereas couch potatoes will judge him to be highly fit.

Finally, researchers have examined not only the content of self-concepts, but their clarity. People with clearer self-concepts respond to questions about themselves more quickly, extremely, and confidently, and their self-concepts are more stable over time (Campbell, 1990). Recent research has pointed to social influences on self-concept clarity. For example, clarity of self-concepts regarding particular traits depends in part on how observable those traits are to others (Stinson et al., 2008b). And when people with low self-esteem (LSEs) receive more social acceptance than they are accustomed to, they become less clear in their self-concepts; the same is true when people with high self-esteem encounter social rejection (Stinson et al., 2010). In sum, social factors are as relevant to understanding the operation of self-concepts as are factors involving the operation of mental representations in individual minds.

Moving to the level of neural mechanisms provides a way of seeing how concepts can function in *all* the ways that psychologists have investigated—as prototypes, exemplars, and theories, if concepts are understood as patterns of neural activity (Thagard, 2010, p. 78),

Simulations with artificial neural networks enable us to see how concepts can have properties associated with sets of exemplars and prototypes. When a neural network is trained with multiple examples, it forms connections between its neurons that enable it to store the features of those examples implicitly. These same connections also enable the population of connected neurons to behave like a prototype, recognizing instances of a concept in accord with their ability to match various typical features rather than having to satisfy a strict set of conditions. Thus even simulated populations of artificial neurons much simpler than real ones in the brain can capture the exemplar and prototype aspects of concepts.

It is trickier to show how neural networks can be used in causal explanations, but current research is investigating how neural patterns can be used for explanatory purposes (Thagard and Litt, 2008). Blouw et al. (forthcoming) present a detailed model of how neural populations can function as exemplars, prototypes, and rule-based explanations.

Another advantage of moving down to the neural level is that it becomes easier to apply multimodal concepts such as ones concerned with physical appearance. People who think of themselves as thin or fat, young or old, and quiet or loud, are applying to themselves representations that are not just verbal but also involve other modalities such as vision and sound. Because much is known about the neural basis of sensory systems, the neural level of analysis makes it easier to see how human concepts can involve representations tied to sensory systems, not only for objects such as cars with associated visual and auditory images, but also for kinds of people (Barsalou, 2008).

Brain scanning experiments reveal important neural aspects of self-concepts. Tasks that involve reflecting on one’s own personality traits, feelings, physical attributes, attitudes, or preferences produce preferential activation in the medial prefrontal cortex (Northoff and Bermpohl, 2004; Mitchell, 2009; Jenkins and Mitchell, 2011). Neural correlates of culturally different self-construals have also have been demonstrated. When East Asian participants were primed with an independent self-construal, right ventrolateral PFC (prefrontal cortex) activity was more active for their own face relative to a coworker’s face, whereas when primed with an interdependent self-construal, this region was activated for both faces (Sui and Han, 2007).

Once concepts are understood partly in neural terms, the relevance of molecular mechanisms becomes evident too, because of the important role of affect and emotion in self-concepts. For most people, thinking of themselves as young and thin carries positive affect, whereas thinking of themselves as old and fat carries negative valence. When such valences are interpreted neurologically, molecular mechanisms involving neurotransmitters and hormones can be applied. For example, the pleasurable feelings associated with *young, thin*, and other concepts that people enjoy applying to themselves plausibly result from activity in neural regions rich in the neurotransmitter dopamine, such as the nucleus accumbens. On the negative side, negative feelings such as anxiety are associated with activity in the amygdala, whose neurons have receptors for the stress hormone cortisol as well as various neurotransmitters. Hence if we want to understand why people much prefer to apply some concepts to themselves and different concepts to others, it is helpful to consider the molecular mechanisms that underlie emotion as well as social, individual, and neural mechanisms. Of course, merely knowing about physiological correlates does not provide causal explanations, which requires mechanisms that link physiology to behavior.

Self-concepts illustrate complex interactions among multiple levels, belying oversimplified reductionist views that see causality as only emanating from lower to higher levels. For example, a social interaction such as a job interview can have the psychological effects of applications of particular concepts (e.g., *nervous* or *competent*) to oneself. Activation of these concepts consists of instantiation of patterns of firing in neural populations, attended by increases and decreases in levels of various chemicals such as cortisol and dopamine. Changes in chemical levels can in turn lead to social changes, as when high cortisol makes a person socially awkward, producing counterproductive social interactions that then lead to self-application of negative concepts. Under such circumstances, the four levels can provide an amplifying feedback loop, from the social to the neuromolecular and back again.

### Self-Presentation (Representing Oneself to Others)

The modes of self-representing discussed so far largely concern how one thinks about oneself, although some aspects of self-image and self-identity also sometimes concern how one wants others to think about oneself. Self-presentation is the central phenomenon for representing oneself to others. It has been discussed extensively by sociologists such as Goffman (1959) and by social psychologists (Leary and Kowalski, 1990). We want to show that self-presentation involves multilevel interacting mechanisms.

Thirty years of research by social psychologists highlight the interplay of the individual and social levels in self-presentation (Schlenker, 2003). One’s goals, at the individual level, affect the social level. People have a basic need for relatedness, for belonging to groups of people that they care about (Baumeister and Leary, 1995; Deci and Ryan, 2000). People know that they are more likely to be accepted by others who have a positive impression of them, so it is natural that people typically want to create a favorable impression. However, people’s goals sometimes lead them to present themselves in socially undesirable ways (for references, see Schlenker, 2003). They may self-deprecate to lower others’ expectations, or try to appear intimidating to generate fear.

The social level also affects the individual level. One’s audience influences one’s self-presentation goals. For example, people tend to be more self-aggrandizing with strangers and more modest with friends (Tice et al., 1995). Particularly striking evidence of the social level affecting the individual level comes from studies indicating that one’s self-presentation to others can influence one’s private self-concept (see Schlenker, 2003; Tice and Wallace, 2003). For example, in one study, participants who had been randomly assigned to present themselves as extraverted were more likely than those who had presented themselves as introverted to later rate themselves as extraverted, and even to behave in a more outgoing fashion, by sitting closer and talking more to others (Fazio et al., 1981). Such self-concept change does not seem to occur unless one’s actions are observed by others (Tice and Wallace, 2003), which again emphasizes the social level. In reviewing the self-presentation literature, Baumeister (1998, p. 705) stated:

People use self-presentation to construct an identity for themselves. Most people have a certain ideal image of the person they would like to be. It is not enough merely to act like that person or to convince oneself that one resembles that person. Identity requires social validation.

Self-presentation is also dependent on neural mechanisms. People naturally fear not being accepted by others, and a variety of studies have found that the social pain of rejection involves some of the same brain areas as physical pain, such as the periaqueductal gray (MacDonald and Leary, 2005). On the other hand, being accepted by others produces pleasure, which involves activation of brain areas such as the nucleus accumbens (Ikemoto and Panksepp, 1999). Izuma et al. (2009) found that the prospect of social approval activates the ventral striatum, which includes the nucleus accumbens. Of course, these neural processes are also molecular ones, with dopamine and opioids associated with positive social experiences, and stress hormones like cortisol associated with negative ones. For example, when people have to give a public speech, often a painful instance of self-presentation, their cortisol levels increase, which may even produce behaviors that undermine the effectiveness of their attempts to produce a good impression (Al’Absi et al., 1997).

Another substance at the molecular level that is likely to be involved in self-presentation is oxytocin, a neuropeptide that has been linked to various social behaviors (e.g., Carter, 1998). Oxytocin is implicated when successful self-presentation requires accurately “reading” other people to understand what would impress or please them, because oxytocin has been linked with social recognition (Kavaliers and Choleris, 2011), empathic accuracy (Rodrigues et al., 2009; Bartz et al., 2010), the processing of positive social cues (Unkelbach et al., 2008), and discerning whether others are trustworthy and should be approached or not (Mikolajczak et al., 2010). Thus, self-presentation involves the complex interaction of social, individual, neural, and molecular mechanisms.

### Self-Esteem (Evaluating Oneself)

The third major kind of self-representing is self-evaluation, which can involve processes such as self-appraisal and self-monitoring, and result in products that range from self-love to self-loathing. We discuss self-esteem as a sample product.

Self-esteem refers to one’s overall evaluation of and liking for oneself. People differ from each other in their characteristic levels of self-esteem, which remain quite stable over time, yet people also fluctuate in their self-esteem around their own average levels. “State self-esteem” refers to one’s feelings about oneself at the moment. Measures of explicit self-esteem obtained by surveys may differ from measures of implicit self-esteem, which are thought to be based associations that are unconscious, or at least less cognitively accessible (Zeigler-Hill and Jordan, 2011).

At the individual level, self-esteem involves the application of self-concepts with positive or negative emotional valence, for example thinking of oneself as a success or failure in important pursuits such as love, work, and play. When people focus on positive aspects of themselves, their state self-esteem increases (e.g., McGuire and McGuire, 1996).

Considerable evidence indicates that social experiences are central to both trait and state self-esteem. According to attachment theory, people begin to learn about their self-worth as infants, in their interactions with caregivers. If the caregiver is loving and responsive to the infant’s needs, the infant develops a model of the self that is worthy of love and responsiveness. If not, the child will develop negative self-models and be anxious in relationships (e.g., Holmes et al., 2005). We have already discussed how social comparisons can influence one’s self-concept; comparisons with other people also can boost or deflate one’s self-esteem (Wood, 1989).

Social acceptance may be the chief determinant of self-esteem. Leary’s sociometer theory proposed that the very existence of self-esteem is due to the need to monitor the degree to which one is accepted and included by other people (Leary and Baumeister, 2000). Indeed, the more people feel included by other people in general, as well as accepted and loved by specific people in their lives, the higher their trait self-esteem (Leary and Baumeister, 2000). Numerous experimental studies indicate that rejection leads to drops in state self-esteem (e.g., Wood et al., 2009a). Interpersonal stressors in the everyday lives of university students are associated with declines in state self-esteem (Stinson et al., 2008a). In contrast, being in a long-term relationship with a loving partner can raise the self-esteem of people with low self-esteem (Murray et al., 1996).

The connection between the individual and social levels of self is also evident in research on how individuals’ self-esteem-related goals influence their social lives. A vast social psychological literature reveals that motivations to maintain, protect, or improve self-esteem can, for example, guide how people present themselves to others (e.g., Baumeister et al., 1989), lead people to compare themselves with others who are less fortunate so as to boost their own spirits (Wood et al., 1985), and lead them to stereotype other people in order to feel better about themselves (Fein and Spencer, 1997; Sinclair and Kunda, 2000).

We have repeatedly described the neural and molecular underpinnings of self-representations involving emotions, and the account of self-concepts as patterns of neural activity associated with particular kinds of neurochemical activity applies directly to self-esteem. Self-esteem is connected with depression, which has been examined at the neural level. Depression and self-esteem are substantially inversely correlated (e.g., with *r*s reaching –0.60 and –0.70 s; Watson et al., 2002); low self-esteem is even one of the symptoms of depression. Depression is well known to have neurotransmitter correlates and to be associated with brain changes.

Evidence is mounting that social acceptance and rejection are accompanied by changes at the neural level (e.g., Eisenberger et al., 2003, 2007; Way et al., 2009). For example, in one study, participants underwent functional magnetic resonance imaging (fMRI) while they viewed words (e.g., boring, interesting) that they believed to be feedback from another person. The rejection-induced drops in self-esteem that we described earlier were accompanied by greater activity in rejection-related neural regions (dorsal ACC, anterior insula; Eisenberger et al., 2011). Neuroimaging studies suggest that the social pain caused by rejection involve the same brain areas as does physical pain (namely, dorsal ACC activity), whereas signs of social acceptance have been associated with subgenual ACC activity (Somerville et al., 2006), and ventral striatum activity (Izuma et al., 2008), neural regions associated with reward (see Lieberman, 2010).

Social threats not only lead to changes in the neural level, but also elicit a host of physiological responses, which point to the links between the social and molecular level. Dickerson et al. (2011) reviewed evidence of cardiovascular (e.g., blood pressure, heart rate), neuroendocrine (e.g., cortisol reactivity), and immune (e.g., inflammatory activity) changes, as well as ways in which social threats “influence the regulation of these systems” (p. 799).

Connections between the social level (rejection and acceptance by others) and the neural level (anterior cingulate and medial prefrontal cortex) have also been associated with the individual level (self-esteem). People who were low in self-esteem differed in their neural responses from those high in self-esteem when others evaluated them (Somerville et al., 2010) or others excluded them (Onoda et al., 2010).

Similarly, individual differences in traits that have been associated with trait self-esteem, rejection sensitivity and attachment styles, have also been linked with differences in neural responses to rejection. Burklund et al. (2007) found that rejection-sensitive people had increased dorsal anterior cingulate activity in response to disapproving facial expressions. Zayas et al. (2009) found that women who differed in attachment styles (which are associated with self-esteem) differed in their neural responses to partner rejection, as reflected in event-related potentials. There is some evidence that the causes of low self-esteem may be genetic as well as social (Roy et al., 1995; Neiss et al., 2002), which provides another reason for moving down to the molecular level in order to consider how genes affecting neural processing might be involved in self-esteem. The operation of the molecular level also may underlie self-esteem differences in responses to stress. Taylor et al. (2003) found that people who had positive self-appraisals had lower cardiovascular responses to stress, more rapid cardiovascular recovery, and lower baseline cortisol levels than people with negative self-appraisals. Furthermore, additional research by Taylor et al. (2008) links these findings with the neural level. Participants with greater psychosocial resources, including higher self-esteem along with other characteristics such as optimism and extraversion, exhibited lower amygdala activity during threat regulation, which appeared to account for their lower cortisol reactivity (Taylor et al., 2008). These psychosocial resources appear to be linked with the oxytocin receptor gene (Saphire-Bernstein et al., 2011).

The interplay of three levels—social, individual, and molecular—is suggested by research by Stinson et al. (2008a). Two studies of university students yielded evidence consistent with their prediction that low self-esteem (individual level) led to interpersonal problems (social level), which in turn resulted in health problems (e.g., missed classes due to illness and visits to the physician). Health problems indicate changes at the molecular level as they imply physiological changes. Dickerson et al. (2011) have made a compelling case that the physiological responses brought about by social threats can worsen physical health.

Considering self-esteem at the neural and molecular levels may provide explanations for why self-esteem in some individuals is less influenced by life experience than learning theories would explain. For example, not all successful people have high self-esteem (e.g., Baumeister et al., 2003a), and it is possible that the exceptions may arise from underlying neural and molecular differences that the individual level does not capture.

## The Effecting Self

In addition to the dozens of self-phenomena concerned with self-representation, there are many phenomena concerned with the self attempting to modify its own states and behavior. These self-effecting phenomena fall into two groups, self-facilitating cases in which one attempts to foster positive aspects of oneself, and self-limiting cases in which one attempts to prevent the behavioral expression of negative aspects of oneself. We will discuss self-enhancement as an important kind of self-facilitation, and self-regulation as an important kind of self-limitation.

### Self-Enhancement

Self-enhancement, the motive to develop and maintain a positive self-view, has been a dominant topic in the social psychological literature for decades. Self-enhancement has been seen as a motivation guiding much of human behavior, with some researchers concluding that it is *the* paramount self-related motive, overriding other goals such as self-accuracy and self-consistency (e.g., Baumeister, 1998; but see Kwang and Swann, 2010). However, a wealth of self-verification studies have provided compelling evidence that people also want to confirm their self-views and to get others to see them as they see themselves (Swann, 2012). Hence self-verification can sometimes be self-limiting and sometimes self-facilitating.

Research has identified many strategies of self-enhancement. To cope with failure, for example, people may attribute the failure externally (e.g., say the test is unfair), minimize the failure, focus on other positive aspects of themselves, derogate other people, or make downward comparisons—that is, compare themselves with others who are inferior (e.g., Blaine and Crocker, 1993; Dodgson and Wood, 1998). Over and over again, research has found that the people who engage in such self-enhancement strategies are dispositionally high in self-esteem, rather than low in self-esteem (e.g., Blaine and Crocker, 1993). This self-esteem difference may occur because people with high self-esteem are more motivated than people with low self-esteem to repair unhappy moods (Heimpel et al., 2002); or because HSEs are more motivated than LSEs to feel good about themselves (e.g., Baumeister et al., 1989); or because LSEs are equally motivated to self-enhance, but cannot as readily claim or defend a positive view of themselves (e.g., Blaine and Crocker, 1993).

One self-enhancement strategy deserves mention because it is a mainstay of self-help books and the popular press: positive self-statements. People facing a stressor, cancer patients, and people chronically low in self-esteem are encouraged to say to themselves such things as, “I am a beautiful person” and “I can do this!” Despite the popularity of positive self-statements and the widespread assumption that they work, their effectiveness was not subjected to scientific scrutiny until recently. Wood et al. (2009b) found that repeating the statement, “I am a lovable person” improved people’s moods only for those who already had high self-esteem. For people with low self-esteem, the statement actually backfired, worsening their moods and their feelings about themselves.

A strikingly different self-enhancement strategy is “self-affirmation” (Steele, 1988). As studied by social psychologists, self-affirmation does not refer to saying positive things to oneself, but to much more subtle methods involving the expression of one’s values. Self-affirmation strategies have included writing a paragraph concerning a value one cherishes (e.g., politics, social connections), or even merely completing a scale highlighting such values. Such strategies seem to be self-enhancing in that they reduce defensiveness (e.g., Crocker et al., 2008), reduce stereotyping (Fein and Spencer, 1997), make people more open to self-evaluation (Spencer et al., 2001), and can substitute for other methods of self-enhancement (e.g., Wood et al., 1999).

Although self-enhancement may seem to be a private matter, operating at the individual level, the social level is clearly influential. Most threats to self-esteem arise in social contexts when feedback from others or others’ behavior leads people to doubt their preferred view of themselves, or to feel devalued or rejected. Hence self-enhancement results from the process of self-evaluation, whose social causes and context we have already discussed. In addition, self-enhancement processes may enlist the social level. Some of the self-enhancement strategies identified above, such as downward comparisons and derogating other people, involve using the social realm to boost oneself at the individual level. Another example comes from research on the triggers of stereotyping. Fein and Spencer (1997) showed that after they fail, people were especially likely to seize on a stereotype of Jewish women. Similarly, after receiving negative feedback, people derogated the person who delivered the feedback, if that person was a woman rather than a man (Sinclair and Kunda, 2000). Other social strategies of self-enhancement can include being boastful and overconfident (e.g., Colvin et al., 1995), helping others (e.g., Brown and Smart, 1991), and aggressing against others (Twenge and Campbell, 2003). People may also enhance themselves through their group memberships and social identities (Banaji and Prentice, 1994). Self-enhancement research, then, reveals links between the individual and social levels of self because the social world often elicits the need for self-enhancement, and certain self-enhancement strategies involve the interpersonal realm. In addition, because self-enhancement can encourage or diminish stereotyping, aggression, and prosocial behavior, self-enhancement clearly has many potential social consequences.

That self-enhancement also operates at the molecular level is shown by a study of self-affirmation. Participants who engaged in a values-affirmation task before they faced a stressor had lower cortisol responses to stress than did participants who had not engaged in values-affirmation (Creswell et al., 2005).

Self-enhancement also operates at the neural level as it involves applications of concepts such as *loveable* which, as we argued earlier, can be understood as patterns of activation in populations of neurons. The study by Wood et al. (2009a) showed that self-statements can alter positive and negative moods, which plausibly involves alteration of activities of neurotransmitters such as dopamine. Better understanding of the neural and genetic determinants of low self-esteem could provide the basis for explaining why positive self-statements can have negative effects on people with low self-esteem.

### Self-Regulation

Although self researchers were long preoccupied with the topics of self-concept and self-esteem, they have come to appreciate that “self-regulation is one of the most important functions of the self” (Gailliot et al., 2008, p. 474). Self-regulation concerns how people pursue their goals or try to control their own behavior, thoughts, or feelings. An idea discussed earlier in the section on self-evaluation—that people continually compare themselves with standards—is central to many theories of self-regulation (e.g., Carver and Scheier, 1990). Such theories posit that when people experience a discrepancy between a standard and their own standing (behavior, thoughts, or feelings) on the relevant dimension, they deliberately or even automatically attempt to reduce that discrepancy, in one of three ways. They can try to adjust their behavior (or thoughts or feelings) so that it meets the standard, change their standards, or exit the situation. Self-regulation is successful when the discrepancy is eliminated or reduced (e.g., Carver and Scheier, 1990).

The biological aspects of the self are most obvious in the self-limiting phenomena aimed at controlling or managing excessive desires for food, alcohol, drugs, sex, or inactivity. Such desires are all rooted in neural and molecular mechanisms that must be counteracted in order to overcome self-destructive behaviors such as overeating. We will not attempt a comprehensive account of all the phenomena concerned with limiting the self, but discuss three main foci of self-regulation research in recent years: goal pursuit, emotion regulation, and ego-depletion—how exercising self-control in one domain diminishes one’s capacity to do so in a second domain.

Research on social comparison establishes a basic connection between the individual and social levels. To meet such goals as self-evaluation, self-improvement, and self-enhancement, individuals compare themselves with other people (Wood, 1989). In this case, other people serve as the standards for meeting one’s goal progress.

Other people can even influence which goals we adopt. Fitzsimons and her colleagues have found that observing a stranger’s goal-directed behavior can lead people to pursue the same goals themselves, or to synchronize their goal pursuits with others, with interesting consequences. For example, people who observe others fail work harder, and people who observe others succeed take it easy (McCullough et al., 2010). Even being in the presence of someone who was a stranger a few minutes before, but who shares similarities such as tastes in movies, can lead one to adopt the other’s goals as one’s own (Walton et al., 2012). Such effects can even occur subconsciously. For example, when participants who had a goal to achieve to please their mother were primed with their mother, they outperformed control participants on an achievement task (Fitzsimons and Bargh, 2003).

One’s own goals also affect one’s relationships with others. People draw closer to others who are instrumental in helping them to progress toward their goals, and distance themselves from others who do not promote such progress (Fitzsimons and Shah, 2008). People seem to cultivate a social environment for themselves that promotes their goals, especially when their progress toward their goals is poor (Fitzsimons and Fishbach, 2010).

Regulation of emotions is an important topic in clinical, social, and cross-cultural psychology (Vandekerckhove et al., 2008). Research on emotion regulation—which concerns how people try to manage their emotional states—has amply demonstrated the interplay between the individual and social levels. For example, people try to adjust their moods in preparation for an upcoming social interaction, according to the social requirements expected (Erber and Erber, 2000). In addition, social events affect one’s emotion regulation: Rejection experiences appear to lead people with low self-esteem to feel less deserving of a good mood, which in turn dampens their motivation to improve a sad mood (Wood et al., 2009a).

A specific example of emotion regulation, anger management, shows the need for multilevel explanations. The strategies for anger management recommended by the American Psychological Association (APA, 2012) operate at all four levels: social, individual, neural, and molecular. Social strategies including expressing concerns with a sympathetic person and moderately communicating with the sources of anger. Humor involving pleasant social interactions can be a potent way of defusing anger. Temporary or permanent removal from anger-provoking social environments can also be helpful.

Psychological strategies for managing anger include the revisions of beliefs, goals, and attitudes. Cognitive therapy aims to help people by changing dysfunctional thinking, behavior, and emotion. Dysfunctional aspects of anger can be addressed by examining whether the beliefs and goals that underlie angry reactions are inaccurate and modifiable. According to the theory of emotions as cognitive appraisals, anger is a judgment that someone or something is thwarting one’s goals, so that anger should be reduced by realization either that the goals are not so important or by revision of beliefs about whatever is thought to be responsible for goal blocking.

Emotions such as anger, however, are not merely cognitive judgments, but also simultaneously involve brain perception of physiological states (Thagard, 2006; Thagard and Aubie, 2008). Hence it is not surprising that anger management techniques include various methods for reducing physiological arousal, such as exercise and relaxation through deep breathing, mediation, and muscle tensing and release. Reducing physiological arousal reduces perception of body states performed by the insula and other brain areas, thereby reducing the overall brain activity that constitutes anger. Similarly, when oxytocin is administered to couples discussing a conflict, their positive verbal and non-verbal behaviors increase (Ditzen et al., 2009).

In severe cases of anger, pharmaceutical treatments may be useful, including anti-depressants such as Prozac that affect the neurotransmitter serotonin, anti-anxiety drugs that affect the neurotransmitter GABA (gamma-Aminobutyric acid), and sometimes even anti-psychotics that affect various other neurotransmitters. The onset of anger can also be exacerbated by recreational use of drugs such as alcohol whose effects on brain chemistry are well known. Hence anger management is an aspect of self-regulation that operates at the molecular level as well as the higher ones.

Ego-depletion studies demonstrate that when people override their emotions, thoughts, impulses, or automatic or habitual behaviors, they have trouble doing so a second time (Baumeister et al., 2007). For example, in one study, research participants had to resist freshly-baked chocolate-chip cookies; they were allowed to eat only radishes instead. When they then faced an impossible puzzle, they gave up more rapidly than participants who had not been required to resist the tempting cookies (Baumeister et al., 1998). In another study, participants who were asked to suppress certain thoughts subsequently had more trouble resisting free beer than did control participants, even when they expected to take a driving test (Muraven et al., 2002).

Ego-depletion research has shown connections between the individual and social levels in two ways. First, difficult social interactions can deplete one’s self-regulatory resources (Vohs et al., 2005). Interracial interactions, for example, can be taxing if one tries not to appear prejudiced. Richeson and Shelton (2003) found that after prejudiced white participants interacted with a black participant, they performed more poorly on a cognitive control task, compared to participants who interacted with a white participant or participants scoring low in prejudice. Social interactions also can be depleting if one is required to engage in atypical self-presentation, such as being boastful to strangers (Vohs et al., 2005). And in yet another example of the harmful consequences of social rejection, studies have indicated that it too can impair self-regulation (see Gailliot et al., 2008, for references).

Second, ego-depletion makes it difficult to navigate social interactions. Participants who had engaged in previous acts of unrelated effortful self-regulation later were more egotistical in their self-descriptions and less able to choose topics for discussion with a stranger that were appropriate in their level of intimacy (Vohs et al., 2005). Self-regulatory depletion also may encourage sexual infidelity and acts of discrimination (Gailliot et al., 2008). Successful self-regulation, then, may smooth one’s interpersonal interactions and make one’s close relationships more harmonious. It is unclear, however, whether ego-depletion is the result of fundamental neural mechanisms of will, or rather individual mechanisms of self-representation: Job et al. (2010) report studies that support the view that reduced self-control after a depleting task or during demanding periods may reflect people’s beliefs about the availability of willpower rather than true resource depletion.

People who have sustained damage to the prefrontal cortex exhibit various self-regulatory deficits, such as impulsivity and poor judgment (see Gailliot et al., 2008, for references). The anterior cingulate is involved in tasks that deplete self-regulatory resources via the coordination of divided attention, and the dorsolateral prefrontal cortex affects the activation, maintenance, and modification of goal-directed responses (Baumeister et al., 2003b). Attempts at self-control recruit a network of brain regions including the lateral and posterior dorsomedial prefrontal cortex (MacDonald et al., 2000). The consensus across thirty neuroimaging studies of emotion regulation in particular is that right ventrolateral PFC and left ventrolateral PFC activity are involved. Other areas also are implicated, including the presupplementary motor area, the posterior dorsomedial PFC, left dorsolateral PFC, and rostral ACC, and their involvement appears to depend on whether the emotion regulation is intentional or incidental to the participants’ task (see Lieberman, 2010, for a review).

Research by Richeson et al. (2003) elegantly links the neural, individual, and social levels of self-regulation. They found that for White participants who held especially negative unconscious attitudes toward Blacks, interacting with a Black person led them to perform poorly on a subsequent self-regulatory task. This effect was mediated by the extent to which these White participants’ dorsolateral prefrontal cortex was activated while they viewed Black faces (in a separate session).

Molecular mechanisms are also undoubtedly involved in self-regulation, although few have been identified. Blood glucose has been thought to underlie ego-depletion phenomena (Gailliot et al., 2008), but recent evidence has challenged that idea (e.g., Molden et al., 2012). Oxytocin may well promote self-regulation in the interpersonal sphere. It appears to lead mothers to tend to their offspring (Taylor, 2002; Feldman et al., 2007), lead people in general to seek and provide social support in stressful circumstances (Taylor, 2002), and to promote helping behavior (Brown and Brown, 2006).

In sum, self-effecting phenomena such as self-enhancement and self-regulation are best understood at multiple mechanistic levels.

## The Changing Self

Self-effecting phenomena involve local changes and behavior, but there is a final group of phenomena that involve more permanent changes to the self (Brinthaupt and Lipka, 1994). We cover two change phenomena: self-expansion and self-development.

### Self-Expansion

According to Aron’s self-expansion theory, human beings have a central desire to expand the self—to acquire resources, perspectives, and identities that enhance their ability to accomplish goals. Self expansion is a motivation to enhance potential efficacy (Aron et al., 2004, p. 105).

This motivation to self-expand at the individual level influences the social level: Aron et al. (2004) argue that self-expansion motives lead people to enter and maintain close relationships with others. In close relationships, each partner includes the other in the self, meaning that each takes on the other’s resources, perspectives, and identities to some extent. Evidence for such processes is illustrated by findings of a study by Aron et al. (1995), who asked university students to respond to the open-ended question “Who are you today?” every 2 weeks for 10 weeks. When respondents had fallen in love during the preceding 2 weeks, their answers to this question revealed increases in the diversity of their self-concept, compared to periods when they had not fallen in love and compared to other respondents who had not fallen in love. They also showed increased self-efficacy and self-esteem. These results remained significant even after mood changes were controlled statistically.

Falling in love also seems to be accompanied by changes in the brain. fMRI studies show that when people who have recently fallen intensely in love look at a photo of or think about their beloved, they have increased activity in the caudate nucleus, which is a central part of the brain’s reward system, as well as in the right ventral tegmental area, a region associated with the production and distribution of dopamine to other brain regions (Aron et al., 2005). Even subliminal priming with a beloved’s name has similar effects (Ortigue et al., 2007). These results suggest that passionate romantic love is associated with dopamine pathways in the reward system of the brain. These dopaminergic pathways are rich in oxytocin receptors (Bartels and Zeki, 2004; Fisher et al., 2006). When women talk about a love experience, oxytocin release is associated with the extent to which they display affiliation cues such as smiles and head nods (Gonzaga et al., 2006).

Recent research offers exciting evidence of possible brain changes with self-expansion. Ortigue and Bianchi-Demicheli (2010) found that when people were primed with their romantic partner’s name (and not a friend’s), they showed more intense activation of the left angular gyrus, the same region that is activated when people think of themselves.

### Self-Development

Self-development refers to the changes that people naturally undergo over the course of their lives. Major developmental periods include early years when infants and toddlers begin to acquire identities (Bloom, 2004; Rochat, 2009), adolescence when teenagers establish increasing independence from parents (Sylwester, 2007), and old age when physical decline imposes new limitations on the self. Each of these periods involves extensive social, individual, neural, and molecular changes, but we will focus on old age, drawing on Breytspraak (1984) and Johnson (2005).

Social relations and the aspects of the self dependent on them change dramatically as people get older. Major changes can include the completion of child-rearing, retirement from employment, diminishing social contacts resulting from physical disabilities, and loss of friends and family to death or infirmity. These changes can all affect the quantity and quality of social interactions that are causally associated with a person’s behaviors and representations.

At the individual level, there are changes in processes, representations, and emotions. Cognitive functioning measured by processing speed and short-term memory capability declines steadily from people’s thirties, and more precipitously in their sixties and later (Salthouse, 2004). Self-conceptions may be stable in some respects, but often alter in others, as people define themselves increasingly in terms of health and physical functioning rather than work roles. People in early stages of old age tend to be happier than those in middle age, but infirmities can bring substantial difficulties (Stone et al., 2010).

Neural causes of changes in the self are most evident in extreme cases like Alzheimer’s disease, when brain degeneration progressively eliminates anything but a minimal sense of self. There are also age-related disorders such as fronto-temporal dementia that can drastically diminish self-effecting phenomena such as self-control (Eslinger et al., 2005). Aging also brings about molecular changes, for example in reduction of levels of hormones such as testosterone and estrogen that affect neural processing. Hence for a combination of social, individual, neural, and molecular reasons, self-development takes on important directions in old age. Similar observations could be made about other crucial stages of personal development such as adolescence. The changing self, like the representing and effecting self, operates through multilevel interacting mechanisms.

## Conclusion

We have shown the relevance of social, individual, neural, and molecular levels to seven important phenomena: self-concepts, self-presentation, self-esteem, self-enhancement, self-regulation, self-expansion, and self-development. These seven are representative of three general classes (self-representing, self-effecting, and self-changing) that cover more than eighty self-phenomena important in psychological discussions of the self.

A full theory of the self will need to specify much more about the nature of the mechanisms at each level, and equally importantly, will need to specify much more about the relations between the levels. Thagard (2014) argued against the common reductionist assumption that causation runs only upward from molecular to neural to individual to social mechanisms. A social interaction such as one person complimenting another has effects on individuals’ mental representations, on neural firing, and on molecular processes such as ones involving dopamine and oxytocin. Fuller explanation of the more than eighty self-phenomena that we have classified in this paper will require elucidation of how they each result from multilevel interactions.

Explanations of complex systems often identify emergent properties, which belong to wholes but not to their parts because they result from the interactions of their parts (Findlay and Thagard, 2012). This basic idea of emergence concerns only the connections of two levels, where the properties of wholes at the higher level (e.g., consciousness) emerge from interactions of parts at the lower levels (neurons). Thinking of the self as resulting from multiple interacting mechanisms points to a more complicated kind of emergence that has gone unrecognized. *Multilevel emergence* occurs when the property of a whole such as the self results from interactions in mechanisms at several different levels, in this case molecular and social as well as neural and cognitive. What you are as a self depends on your genes and your social influences as well as on your semantic pointers and mental representations. Major changes in the self such as religious conversions, dramatic career shifts, and recovery from mental illness are critical transitions that result from interactions among multiple levels. For example, recovery from severe depression often requires (1) changes in neurotransmitters through medication operating at the molecular and neural levels and (2) changes in beliefs and goals through psychotherapy operating at the mental and social levels. Future theoretical work on the self will benefit from more detailed accounts of the interactions of individual, social, neural, and molecular mechanisms.

### Conflict of Interest Statement

The authors declare that the research was conducted in the absence of any commercial or financial relationships that could be construed as a potential conflict of interest.
